# Advances in multiscale myelin imaging: from classical histology to functional insights

**DOI:** 10.3389/fncel.2026.1771168

**Published:** 2026-02-18

**Authors:** Kentaro Okuyama, Yuji Komaki, Motonari Ishihara, Yuma Kurosaki, Saki Tsuchiya, Manabu Hayatsu, Junpei Nakayama, Keiko Uchiyama, Kosuke Itoh, Toshihiro Nagai, Tomoko Shindo, Nobuko Moritoki, Hiroyuki Kawashima, Shinsuke Shibata

**Affiliations:** 1 Division of Microscopic Anatomy, Graduate School of Medical and Dental Sciences, Niigata University, Niigata, Japan; 2 Bioimaging Center, Central Institute for Experimental Medicine and Life Science, Kanagawa, Japan; 3 Division of Orthopedic Surgery, Graduate School of Medical and Dental Sciences, Niigata University, Niigata, Japan; 4 Center for Integrated Human Brain Science, Brain Research Institute, Niigata University, Niigata, Japan; 5 Electron Microscope Laboratory, Keio University School of Medicine, Tokyo, Japan

**Keywords:** electron microscopy, imaging, light microscopy, myelin, non-invasive imaging, oligodendrocytes, schwann cells

## Abstract

Scientific understanding of myelin, the lipid-rich sheath of axons essential for vertebrate rapid neuronal communication, has evolved considerably. Enabled by major advances in imaging technology, research has shifted from viewing myelin as a static insulator to investigating the dynamic roles of myelinating glia in nervous system development, function, and pathophysiology. This review aimed to provide a comprehensive, multi-scale overview of the imaging toolkit for myelin biology, from foundational histology to cutting-edge advances. At the macro- and meso-scales, non-invasive modalities like magnetic resonance imaging and positron emission tomography reveal *in vivo* myelin architecture and molecular changes, offering critical insights into large-scale pathology. At the micro-scale, advanced light microscopy now visualizes cellular dynamics and molecular interactions with remarkable clarity. Finally, at the nano-scale, sophisticated electron microscopy techniques—including volume electron microscopy and correlative approaches—resolve the ultrastructural basis of biological phenomena with unparalleled detail. As no single modality can capture the full biological context, a holistic understanding of glial biology requires the strategic integration of these multi-scale techniques with advanced computational analysis. This integrated approach is essential for revealing the full spectrum of myelin biology and uncovering novel targets for therapeutic intervention.

## 1 Introduction

**FIGURE 1:**
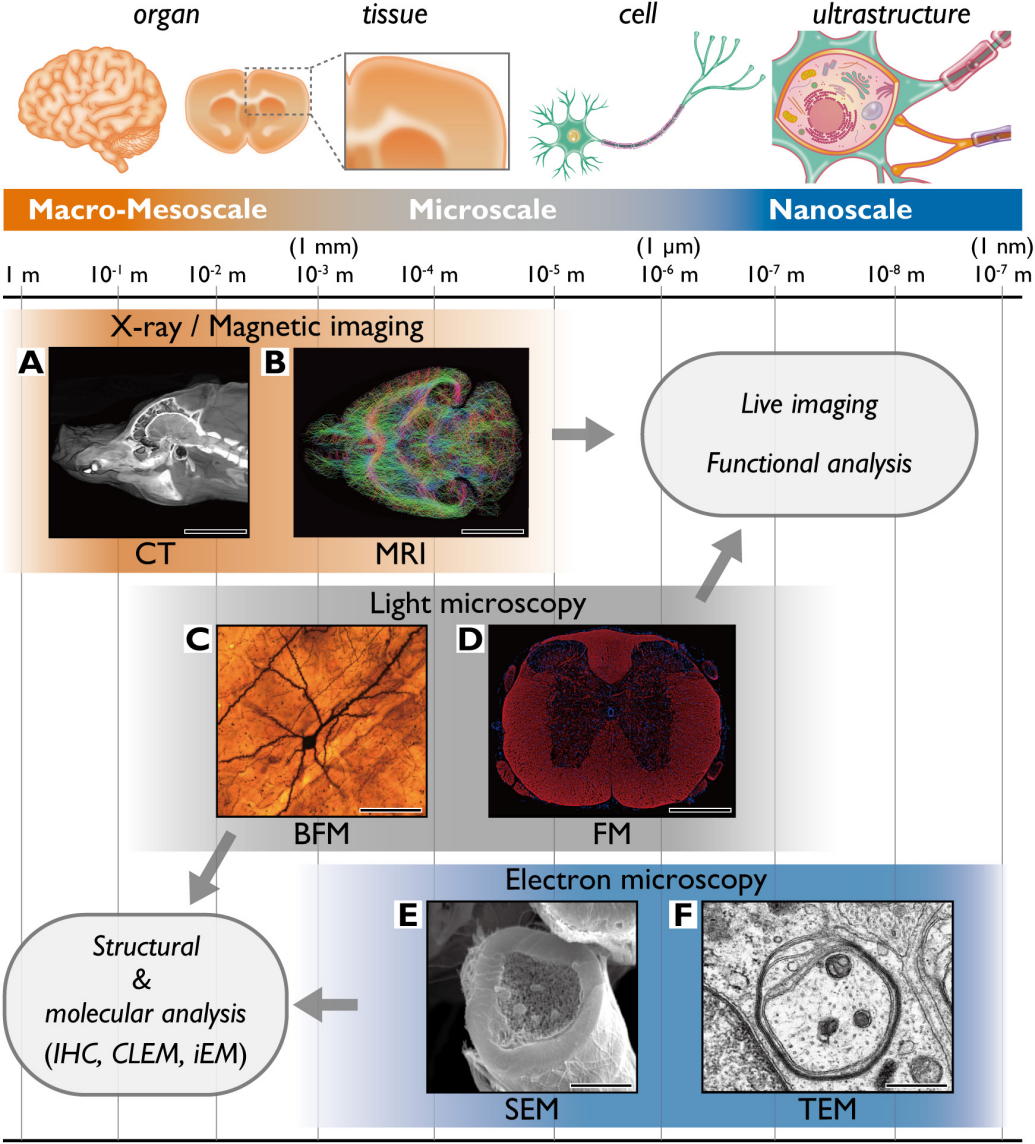
Spatial resolution and target structures of common imaging techniques. **(A)** 3D computed x-ray tomography reconstruction of a pig craniocervical region (lateral view). Scale bar = 10 cm. **(B)** MRI-based myelin tract in a mouse brain (axial view). Scale bar = 4 mm. **(C)** Brightfield microscopy image of a Golgi-stained pyramidal neuron in a rat cerebral cortex. Scale bar = 50 μm. **(D)** Confocal microscopy image showing fluorescently labeled myelin (anti-MBP, red) and nuclei (Hoechst, blue) in a mouse spinal cord. Scale bar = 1 mm. **(E)** SEM image of a myelinated axon in a mouse sciatic nerve. Scale bar = 3 μm. **(F)** TEM image showing the early phase of myelination in a rat sciatic nerve. Scale bar = 500 nm. BFM, brightfield microscopy; CT, computer tomography; CLEM, Correlative light and electron microscopy; FM, fluorescence microscopy; iEM, immunoelectron microscopy; IHC, immunohistochemistry; MRI, magnetic resonance imaging; SEM, scanning electron microscopy; TEM, transmission electron microscopy.

**FIGURE 2:**
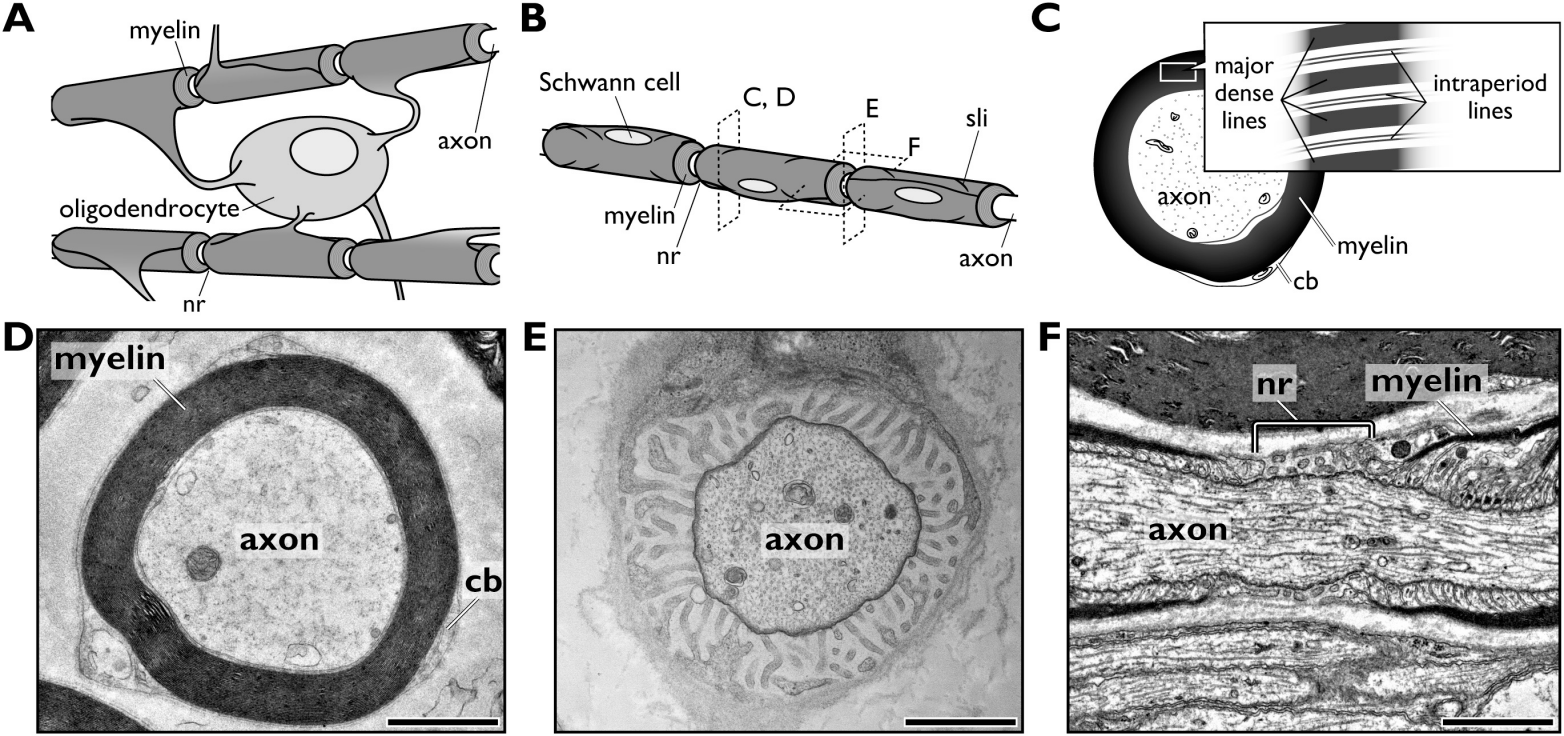
Myelinated axons in the vertebrate nervous systems. **(A,B)** Schematic diagrams comparing myelinated axons in the central nervous system, where a single oligodendrocyte myelinates multiple axons **(A)**, and the peripheral nervous system, where a single Schwann cell myelinates one axonal segment **(B)**. Dashed boxes in panel **(B)** indicate the approximate views shown in panels (**C–F)**. **(C)** Schematic cross-section of a myelinated axon in the PNS, showing the axon, the compacted myelin sheath, and the surrounding Schwann cell cytoplasm. **(D–F)** Transmission electron micrographs of a myelinated axon in a mouse sciatic nerve. **(D)** Cross-section of a myelinated internode. **(E)** Cross-section of a node of Ranvier. **(F)** Longitudinal section of a node of Ranvier. Scale bars = 2 μm. cb, Cajal bands; nr, node of Ranvier; sli, Schmidt–Lanterman incisure.

Myelin is a lipid-rich, multi-lamellar sheath that spirally wraps axons in both the central (CNS) and peripheral (PNS) nervous systems. Its structural integrity is crucial for neural function, making myelin imaging vital for both neurological research and clinical assessment (Figure 1). In the CNS, a single oligodendrocyte myelinates multiple axons (Figure 2A), whereas in the PNS, each Schwann cell typically ensheaths a single axon segment (Figure 2B). The PNS myelin contains unique cytoplasmic structures, including Schmidt–Lanterman incisures and the Cajal bands—parts of the Schwann cell cytoplasm left behind in the sheath. However, the fundamental ultrastructure of compact myelin is conserved across both nervous systems: the “major dense line,” formed by the compacted intracellular surface of the glial cell, and the “intraperiod line,” formed by the apposed extracellular surfaces (Figures 2C,D; Simons and Nave, 2016). Because compact myelin is primarily composed of lipids (70%–80% of weight) and proteins (20%–30%), it acts as an electrical insulator. This insulation is interrupted at the node of Ranvier, small unmyelinated gaps where voltage-gated ion channels cluster (Figures 2A,B,E,F), enabling action potentials to jump between nodes via saltatory conduction. This process dramatically increases signal velocity, establishing myelin as fundamental to rapid signaling in the vertebrate nervous system. Myelin’s high lipid content makes it prone to fixation-induced artifacts, including swelling or shrinkage. Preserving native ultrastructure is crucial, because ultrastructural changes precede overt demyelination and exacerbate neurodegenerative pathology (McNamara et al., 2023; Depp et al., 2023). Thus, achieving artifact-free imaging is a key challenge for neuropathology (Weis et al., 2021). This review aimed to provide a comprehensive overview of myelin imaging, covering general methods, specialized techniques, and recent advances.

## 2 Macro- to meso-scale imaging approaches

### 2.1 Magnetic resonance imaging (MRI)

**FIGURE 3:**
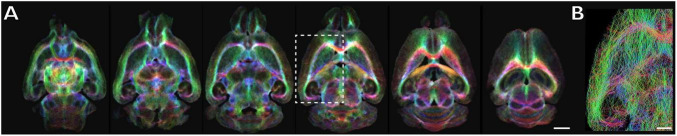
Myelin map of the mouse brain by MR track density imaging. **(A)** Axial TDI (TE 21.5 ms, TR 225 ms, *b*-value 3,000 s/mm^2^, 30 direction (5 B0 images), spatial resolution 100 μm, acquisition time 15 h 59 min) map images of the mouse brain. The TDI map was computed using MRtrix3 based on the following parameters: method, iFOD2; max number of tracks, 4,000,000; voxel size, 20 μm. Scale bar = 2 mm. **(B)** Tractography for the region enclosed by the dashed square in panel **(A)**, with a depth of 3,227 μm from bregma. Scale bar = 1 mm.

Among macro- to meso-scale imaging of nervous tissue, magnetic resonance imaging (MRI) remains the gold standard for non-invasive imaging in both basic research and the pathological evaluation of neurodegenerative diseases (van der Weijden et al., 2021; Paquola and Hong, 2023). To assess axon and myelin integrity, diffusion tensor imaging (DTI) and myelin water imaging/fraction (MWI/MWF) are often employed (Mori and Zhang, 2006; Lee et al., 2021). DTI parameters such as fractional anisotropy (FA) and radial diffusivity (RD) measure water molecule diffusion, providing insight into tissue structural characteristics. Although myelin restricts water diffusion perpendicular to the axon, intact myelinated axons tend to exhibit higher FA and lower RD, whereas demyelinated regions are characterized by reduced FA and increased RD (Basser et al., 1994; Conturo et al., 1999; Takagi et al., 2009; Fujiyoshi et al., 2016). A validation study employing the cuprizone-induced demyelination and remyelination model demonstrated a correlation between DTI parameters and myelin content, with RD emerging as the most sensitive myelin status indicator (Yano et al., 2017). These methods are valuable for myelin imaging owing to their sensitivity to microstructural changes; however, these metrics are not exclusively myelin-specific, as several factors influence their detection, including axonal density, fiber orientation dispersion, and extracellular water content (Friedrich et al., 2020). Conversely, myelin water imaging directly quantifies MWF, estimating the proportion of water trapped between the myelin bilayers relative to the total water signal (Mackay et al., 1994; Whittall et al., 1997), isolating the signal specifically associated with myelin water based on its distinct T2 relaxation time, offering a more myelin-specific measurement than DTI. However, its sensitivity to noise, longer scan times, and complex data processing remain challenging (Uddin et al., 2019; Paquola and Hong, 2023; Xu et al., 2025). Combining DTI’s microstructural sensitivity with MWF’s direct myelin detection provides complementary information. Active development of high-resolution *in vivo* MRI aims to achieve *ex vivo*-level resolutions, improving clinical studies and tractography (Mohammadi and Callaghan, 2021; Baadsvik et al., 2024; Bosticardo et al., 2024; Thompson et al., 2025). Track-density imaging (TDI), a novel MRI technique, produces high-resolution white matter images using diffusion MRI fiber-tracking data (Calamante et al., 2010; Figure 3). This super-resolution method utilizes long-range information from fiber tracks to generate images with enhanced spatial resolution and anatomical contrast beyond the acquired voxel size (Calamante et al., 2011). A voxel—or a “volume element”—represents a discrete unit of data on a three-dimensional (3D) grid, extending the two-dimensional (2D) pixel concept into 3D space (Thaler et al., 1978). Studies comparing TDI maps of *ex vivo* mouse brains with histological staining have shown good agreement, confirming that TDI provides valid anatomical information (Calamante et al., 2012).

Although macro-scale MRI has traditionally been CNS-centric, it is increasingly being applied to the PNS to provide morphological and functional insights (Chen et al., 2019). Advanced MRI metrics, particularly diffusion-weighted imaging and DTI, are applicable to the PNS, offering quantitative biomarkers for nerve fiber organization, axonal flow, and myelin integrity (Martín Noguerol et al., 2017). For instance, diffusion tensor tractography has been used to visualize the degeneration and regeneration of rat sciatic nerves, enabling the precise tracking of fiber recovery (Takagi et al., 2009). However, PNS-specific imaging faces distinct challenges compared to CNS-specific imaging. The small size and complex branching of peripheral nerves require higher spatial resolution; the presence of surrounding adipose tissue necessitates robust fat-suppression techniques. The varying orientation of nerves relative to the main magnetic field (B_0_) can induce “magic angle” effects (Chappell et al., 2004), possibly confounding quantitative metrics—a limitation less prevalent in the more uniformly oriented white matter tracts of the CNS.

### 2.2 Positron emission tomography (PET)

Positron emission tomography (PET) is another *in vivo*, molecular imaging modality that uses radiolabeled tracers targeting myelin-associated proteins, making it effective for evaluating CNS diseases such as multiple sclerosis. Several promising tracers have demonstrated myelin sensitivity (de Paula Faria et al., 2014; Auvity et al., 2020): stilbene derivatives, including [^11^C]N-methyl-4,4’-diaminostilbene (MeDAS), targeting myelin basic protein (MBP) (Wu et al., 2010; van der Weijden et al., 2022, 2025), ^18^F-florbetaben (Matías-Guiu et al., 2015; Pytel et al., 2020), and ^18^F-florbetapir (Carotenuto et al., 2020; Zhang et al., 2021), and thioflavin-T derivatives, including [^11^C]Pittsburgh Compound B (PiB), targeting the beta-sheet of MBP (Stankoff et al., 2011) and its fluorinated derivative, ^18^F-flutemetamol, originally developed as amyloid radioligands but now investigated as potential myelin tracer (Nelissen et al., 2009; Zeydan et al., 2022). PET offers high molecular sensitivity for quantifying myelin integrity but has lower spatial resolution than MRI and involves ionizing radiation, limiting longitudinal studies. Many researchers combine MRI’s anatomical detail with PET’s molecular specificity for a comprehensive assessment of myelin integrity and pathology (Carotenuto et al., 2020; van der Weijden et al., 2023). Regarding PNS application, although several molecular targets such as MBP are conserved, PET imaging is hindered by the small size of peripheral nerves, leading to significant partial volume effects. Additionally, non-specific uptake in surrounding tissues, including muscle and fat tissue, compromises contrast, limiting PET’s utility in peripheral neuropathies compared to that in the CNS. Recently emerging multimodal approaches, including hybrid PET/MRI using amyloid tracers, have demonstrated potential in visualizing peripheral-nerve pathology (Shouman et al., 2021), suggesting that anatomical guidance mitigates these spatial limitations.

### 2.3 X-ray computed tomography (CT)

Recent advancements in X-ray computed tomography (CT) enable non-destructive, micron-scale 3D myelin imaging, overcoming conventional contrast limitations for soft tissue. Small-angle X-ray scattering-CT provides label-free visualization by detecting scattering signals generated by the ultrastructural periodicity of myelin (De Felici et al., 2008; Jensen et al., 2011). This allows the simultaneous quantification of myelin integrity and orientation, but imaging large volumes such as entire human brain remains challenging (Georgiadis et al., 2021). X-ray phase-contrast tomography detects phase shifts for high-contrast whole-brain imaging (Chourrout et al., 2022). This method relies on simple ethanol dehydration rather than on staining to generate contrast, although high-resolution imaging requires limited-access synchrotron sources. Diffusible iodine-based contrast-enhanced CT improves contrast by using iodine to bind tissue lipids, thereby increasing the radiodensity of myelinated regions for high-throughput, 3D visualization of nerve architectures (Gignac et al., 2016; Gignac and Kley, 2018; Balcaen et al., 2023). Limitations of this method include tissue shrinkage, prolonged diffusion times for large specimens, and a lack of myelin specificity owing to non-specific lipid binding (Gignac and Kley, 2018).

## 3 Micro-scale myelin imaging using light microscopies (LMs)

### 3.1 General light and fluorescence microscopic imaging

**FIGURE 4:**
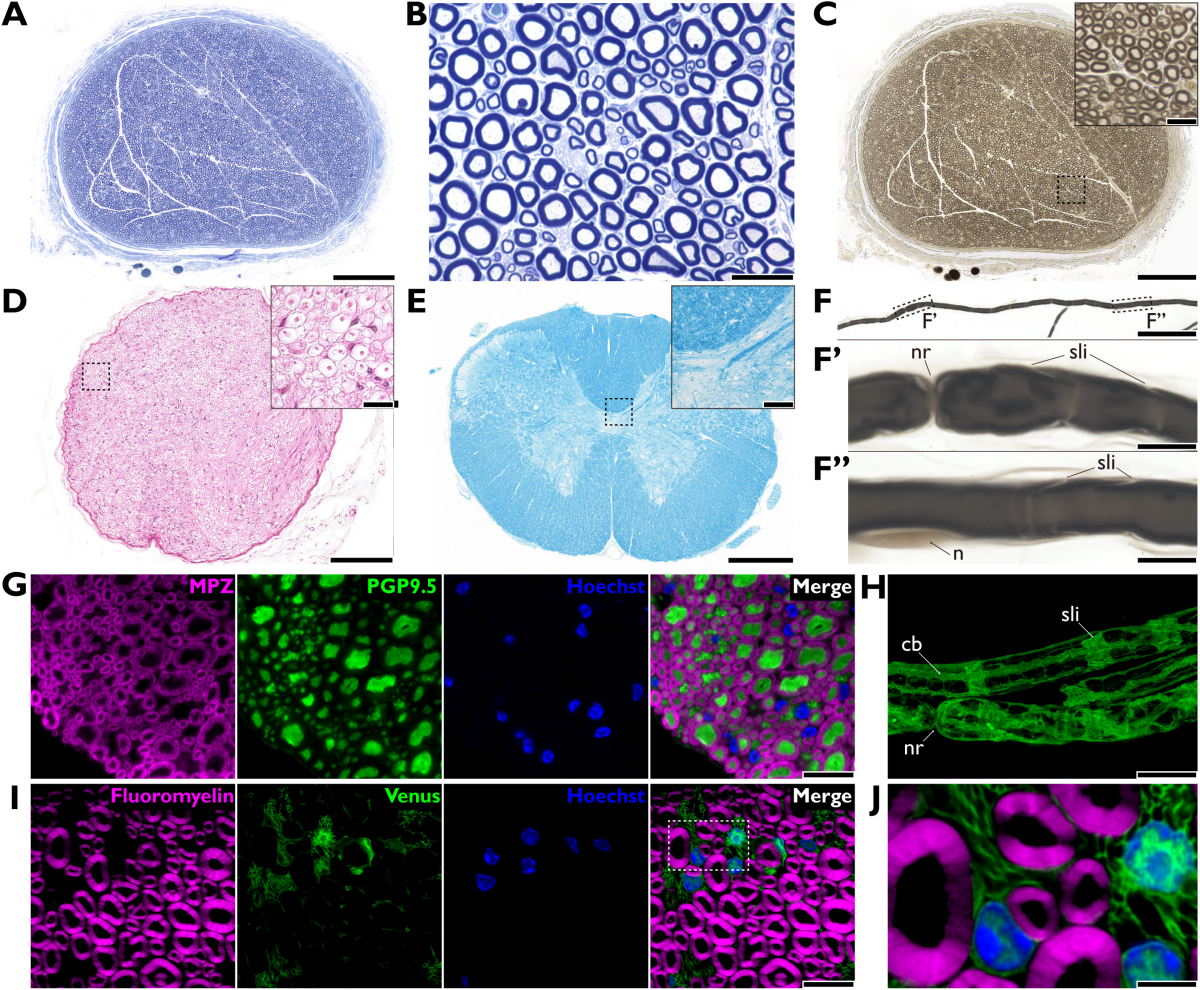
Myelin imaging by light microscopies. **(A,B)** Resin-embedded section of a rat sciatic nerve stained with toluidine blue. An overview **(A)** and a magnified view **(B)**. Scale bars = 200 μm **(A)**, = 20 μm **(B)**. **(C)** Resin-embedded section of a rat sciatic nerve stained with osmium tetroxide during the post-fixation process. The inset shows a magnified view of the dashed square area. Scale bars = 200 μm **(C)**, = 20 μm (inset). **(D)** Paraffin-embedded section of a rabbit sciatic nerve stained with hematoxylin and eosin. The inset shows a magnified view of the dashed square area. Note the artifactual swelling of the myelin sheath, which appears as a void, a common result of lipid loss during processing. Scale bars = 200 μm **(D)**, = 20 μm (inset). **(E)** Paraffin-embedded section of a rat spinal cord stained with Luxol Fast Blue. Myelinated tracts are stained blue. The inset shows a magnified view of the dashed square area. Scale bars = 500 μm **(E)**, =20 μm (inset). **(F)** Single myelinated axon from a mouse sciatic nerve prepared using the teased-fiber method and stained with osmium tetroxide. The dashed square areas **(F’,F”)** in the top panel are shown at higher magnification **(F’,F”)**, respectively. Scale bars = 100 μm **(F)**, =10 μm **(F’,F”)**. **(G)** Fluorescent immunohistochemistry of a mouse dorsal root. Myelin is labeled with anti-myelin protein zero antibody (MPZ; magenta), axons with anti-PGP9.5 antibody (green), and nuclei with Hoechst 33258 (blue). Scale bar = 10 μm. **(H)** Maximum intensity projection from a super-resolution confocal z-stack showing the intrinsic fluorescent signal expressed in cytoplasm of Schwann cells in a Sox10-Venus mouse sciatic nerve, prepared using the teased-fiber method. Scale bar = 10 μm. **(I,J)** Confocal images of an unfixed Sox10-Venus mouse sciatic nerve section. Myelin is stained with Fluoromyelin™ (magenta), Schwann cells express endogenous Venus (green), and nuclei are stained with Hoechst (blue). **(J)** A magnified view of the dashed square region in panel **(I)**. Scale bars = 10 μm **(I)**, = 3 μm **(J)**. cb, Cajal band; n, nuclear; nr, node of Ranvier; sli, Schmidt-Lanterman incisure.

For myelin imaging with LMs, toluidine blue staining of resin-embedded sections (Figures 4A,B) is a common method in research and pathology for neurodegenerative diseases, including amyotrophic lateral sclerosis and multiple sclerosis (Carriel et al., 2017; Ghnenis et al., 2018). In the standard protocol for resin embedding, the myelin sheath structure is well-preserved because glutaraldehyde strongly fixes proteins while osmium tetroxide stabilizes lipids. Osmium tetroxide effectively fixes lipids and stains myelin a dark brown to black (Figure 4C). It can be integrated into standard paraffin-embedding procedures and subsequent staining techniques, including connective tissue staining and immunohistochemistry (IHC) (Scipio et al., 2008). Conversely, conventional hematoxylin and eosin staining for the paraffin-embedded sections may not yield accurate results for myelin morphology observation because the myelin sheath is often swollen or shrunk (inset in Figure 4D) owing to the lipid loss during dehydration, as they are poorly preserved by common formaldehyde-based fixatives like formalin. For paraffin-embedded sections, myelin-specific stains such as Luxol Fast Blue staining (Figure 4E), Sudan Black B staining (Stilwell, 1957; Ineichen et al., 2017), and Klüver–Barrera (a combination of Luxol Fast Blue and Nissl staining) (Klüver and Barrera, 1953), are more frequently employed. Additionally, the haloaurophosphate complex (black gold) (Schmued and Slikker, 1999; Savaskan et al., 2009) and modified Gallyas silver staining (Gallyas, 1979; Pistorio et al., 2006) are suitable for high-resolution imaging of myelinated axons.

The teased-fiber method mechanical dissociates nerve fascicles into individual myelinated axons, enabling observation of continuous longitudinal profiles without sectioning (Figures 4F–F”). While clinically indispensable for differentiating segmental demyelination from axonal degeneration, this method is labor-intensive owing to manual preparation (Bolon et al., 2020). Combined with confocal microscopy, this method facilitates the high-resolution imaging of axon-glia interfaces and cytoskeletal organization at the nodes of Ranvier (D’Este et al., 2017; Sell et al., 2024).

Fluorescence microscopy offers higher contrast and can preserve physiological information (Figures 4G–J). Confocal laser scanning microscopy has demonstrated broad utility for *in vivo* and/or finer imaging and quantitative analysis of biological specimens, including myelin (Reynolds et al., 1994; Beirowski et al., 2004; Schain et al., 2014; Kwon et al., 2017). Conventionally, target structures are fluorescently labeled by IHC or transgenic techniques. Conversely, reflectance imaging, such as spectral confocal reflectance microscopy, exploits optical properties of the multi-layered myelin sheath to generate label-free visualizations based on thin-film interference (Schain et al., 2014; Kwon et al., 2017). This signal generation relies on the tightly periodic structure of compact myelin. The distinct optical signature allows for the spatial differentiation of compact myelin domains from other structural features, specifically the nodes of Ranvier and Schmidt-Lanterman incisures. Because the reflectance signal is highly sensitive to lamellar organization, this modality is particularly effective at detecting pathological changes, such as myelin decompaction or swelling, where the loss of structural integrity results in signal alteration or attenuation (Gonsalvez et al., 2019; Craig et al., 2024). Other advanced LM modalities have been utilized for non-invasive, high-resolution, label-free myelin imaging. These include coherent anti-stokes Raman scattering microscopy, providing contrast by detecting the specific molecular vibrations of C-H bonds abundant in the lipid-rich myelin sheath (Wang et al., 2005; Huff and Cheng, 2007; Freudiger et al., 2008; Poulen et al., 2021); birefringence microscopy that detects the intrinsic birefringence, an optical anisotropy arising from the highly organized lipid bilayer structure of myelin and reflecting its structural integrity and function (Blanke et al., 2021, 2023; Morgan et al., 2021); non-linear LMs such as two-photon excitation microscopy that capture intrinsic tissue autofluorescence (Canty et al., 2013; Costantini et al., 2021; Wu et al., 2021); and second- or third-harmonic generation microscopy, generating signals based on non-centrosymmetric structures or optical heterogeneities at lipid-aqueous interfaces, respectively (Farrar et al., 2011; Lim et al., 2014; Rishøj et al., 2022). Although conventional resolution is limited by diffraction to approximately 0.2 μm, super-resolution imaging systems achieve resolutions below 0.1 μm, enabling more accurate analysis with high-contrast imaging (Figure 4H; D’Este et al., 2017; Coto Hernández et al., 2022b; Chen et al., 2024). Recent advances further complement conventional 2D analysis, enabling higher resolution and larger volume 3D imaging with detailed quantitative evaluation (Velasco et al., 2021; Wu et al., 2022; Tsutsumi et al., 2023). This progress integrates these advanced microscopes with innovative sample preparations, such as tissue clearing, and sophisticated computational processing.

Several LM imaging approaches specifically target myelin. Fluoromyelin*™* is a widely used and rapid dye for labeling myelin lipid components (Figures 4I,J; Monsma and Brown, 2012; Wang et al., 2019). Several fluorescent compounds that cross the blood–brain barrier have been developed for *in vivo* optical imaging, many with potential applications in PET imaging (Wu et al., 2006; Wang et al., 2009, 2010, 2011). Among them, near-infrared probes (Xiang et al., 2005; Wang et al., 2011) enable deeper tissue penetration and higher-resolution *in vivo* imaging with advanced LMs (Wu et al., 2021). The fluorescent probe Nile red can detect polarity changes in myelin lipids (considered early indicators of pathology before demyelination) when combined with spectroscopy (Luchicchi et al., 2021; Teo et al., 2021, 2025). Microscopy with an ultraviolet surface-excitation system utilizes deep ultraviolet light excitation (approximately 280 nm), penetrating tissue only superficially and effectively provides optical sectioning without the need for thin physical slicing (Fereidouni et al., 2017). This system offers rapid, label-free myelin imaging with high-intrinsic contrast, supporting 2D and 3D analyses (Kolluru et al., 2022).

### 3.2 Immunohistochemistry for myelin imaging

Immunohistochemistry is widely used to visualize myelin morphology and profile by localizing molecular markers with tagged antibodies (Figure 4G). This section summarizes several key molecular markers for myelin and myelin-forming glial cells at the LM-level IHC.

The protein constituents of the myelin sheath serve as robust IHC markers. MBP (Omlin, 1982; Barbarese et al., 1988; Werner et al., 2013), 2’3’-cyclic nucleotide 3’phosphodiesterase (CNPase) (Gravel et al., 1997), and myelin-associated glycoprotein (Quarles, 2007) are conserved components of CNS and PNS myelin. In the CNS, the major structural proteins include proteolipid protein (PLP) (Griffiths et al., 1998; Greenfield et al., 2006), myelin oligodendrocyte glycoprotein (MOG) (Scolding et al., 1989), and Claudin-11 (Bronstein et al., 1997; Tiwari-Woodruff et al., 2001), whereas PNS myelin is enriched in myelin protein zero (P0/MPZ) (Eylar et al., 1979). The epitope-specific serology enables discrimination between compact and uncompacted myelin structures. Matsuo et al. (1997) demonstrated that a specific peptide sequence of MBP (residues 82–88 in humans) is structurally masked within healthy compact myelin but becomes exposed during structural loosening or degeneration. Antibodies targeting this specific epitope, which are often referred to as degraded MBP (dMBP), selectively label uncompacted or damaged myelin and differ from conventional anti-MBP antibodies that recognize the total myelin content. This approach provides a sensitive metric for assessing myelin integrity and white matter pathology (Ihara et al., 2010).

Myelinating glia markers are equally important. The oligodendrocyte lineage is defined by stage-specific molecular signatures: pan-lineage identity is established by the transcription factors Olig2 (Takebayashi et al., 2000; Zhou et al., 2000) and Sox10 (Stolt et al., 2002; Sock and Wegner, 2021); oligodendrocyte precursors identified by PDGFRα, the surface antigen NG2 (Nishiyama et al., 1996), and a transcription factor Nkx2.2, regulating early differentiation and is downregulated in the mature state (Qi et al., 2001; Cai et al., 2010); differentiating states are characterized by galactosphingolipid (GalC and O4) expression (Sommer and Schachner, 1981); and mature, myelinating states are characterized by CNPase and CC1 (Bin et al., 2016). The Schwann cell lineage is also well-characterized by state-dependent markers. Sox10 (Kuhlbrodt et al., 1998; Inoue et al., 1999; Britsch et al., 2001) and S100β (Stefansson et al., 1982) serve as canonical markers, while the myelination program is directed by the transcriptional regulator Egr2/Krox20 (Topilko et al., 1994; Decker et al., 2006), driving the expression of integral myelin proteins such as P0 and PMP22 (Snipes et al., 1992; Pareek et al., 1993). Conversely, the non-myelinating phenotype is characterized by high levels of glial fibrillary acidic protein (GFAP) and P75NTR (Yu et al., 2009; Jung et al., 2011). Following nerve injury, cells transform into a repair phenotype, under the control of c-Jun, re-expressing GFAP, P75NTR, and the progenitor-associated factor Sox2 (Jessen and Arthur-Farraj, 2019; Jessen and Mirsky, 2022). Clinically, the immunohistochemical detection of these markers is utilized for analyzing histopathological features in demyelinating diseases such as multiple sclerosis (Greer and Pender, 2008; Stork et al., 2020) and Charcot–Marie–Tooth syndrome (Nadol et al., 2018). Additionally, *in situ* hybridization for specific mRNAs, such as *MBP*, *PLP*, and *MOG*, is used to investigate the state of myelin-forming glial cells (Breitschopf et al., 1992; Michel et al., 2015).

### 3.3 Transgenic mouse strains useful for myelin observation

Although static analyses with IHC are valuable for observing myelin in research and pathological diagnosis, they are often insufficient for elucidating the dynamic regulation of myelination. Transgenic mouse strains expressing fluorescent reporters (such as green fluorescent protein) under cell-specific promoters are powerful tools for visualizing cell morphology and tracking cellular dynamics in real time, significantly advancing our understanding of nervous system function, including myelin biology.

Examples include PLP-EGFP mice for visualizing oligodendrocyte lineage differentiation and myelination (Mallon et al., 2002), CNPase-EGFP mice for detailed myelination observation in the CNS and PNS (Yuan et al., 2002; Deng et al., 2014). Additionally, Sox10-Venus mice exhibit a robust Venus signal, enabling direct imaging of oligodendrocytes and Schwann cells even in live or unfixed tissue (Figures 4H–J; Shibata et al., 2010; Mohan et al., 2019). Furthermore, crossing Cre-driver lines such as Wnt1-Cre (Danielian et al., 1998), P0-Cre (Yamauchi et al., 1999), MBP-Cre (Kolb and Siddell, 1996), PLP-Cre (Doerflinger et al., 2003), and Sox10-Cre (Stine et al., 2009) with Cre-dependent reporter strains (Kawamoto et al., 2000) provides powerful tools for tracing the dynamics of myelinating glial cells.

## 4 Nano-scale myelin imaging using electron microscopies (EMs)

### 4.1 General electron microscopic imaging

**FIGURE 5:**
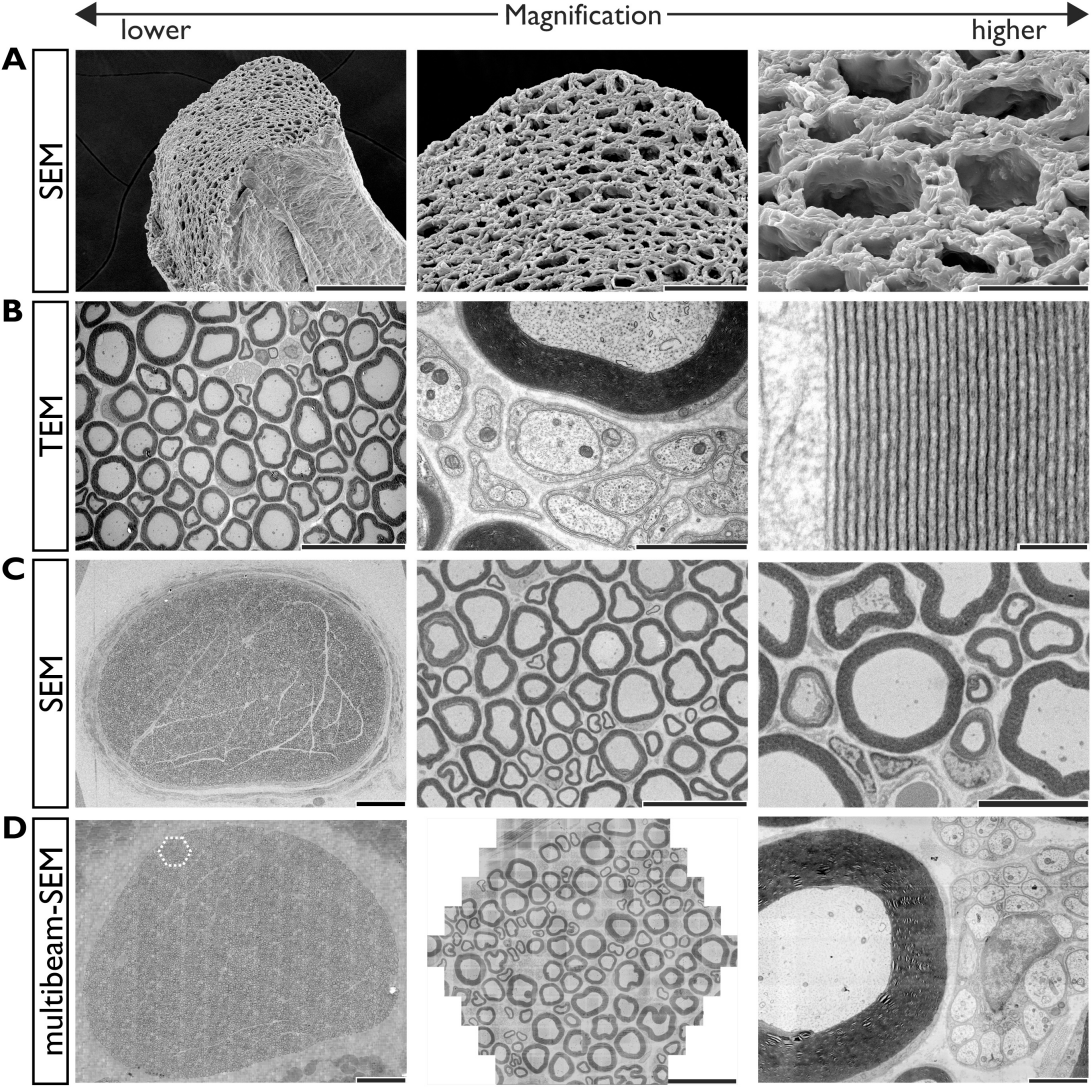
Myelin imaging by electron microscopies. **(A)** SEM images of a mouse dorsal funiculus prepared with the osmium maceration method. A low-magnification oblique view of the tissue **(**left**),** two progressively higher-magnification views **(**middle and right**)**. Scale bars = 100 μm (left), = 30 μm (middle), = 10 μm (right). **(B)** TEM images of a mouse sciatic nerve ultrathin section. A low-magnification view (left), a higher-magnification view of myelinated and unmyelinated axons (middle), and a high-magnification view of the myelin lamellae (right). Scale bars = 30 μm (left), = 2 μm (middle), = 0.1 μm (right). **(C)** SEM images of a rat sciatic nerve ultrathin section, imaged using a backscattered electron detector (signal inverted). A low-magnification overview of the entire section (left) and two progressively higher-magnification views (middle and right). Scale bars = 200 μm (left), = 30 μm (middle), = 10 μm (right). **(D)** Multibeam SEM images of a rat sciatic nerve ultrathin section, imaged using a secondary electron detector (signal inverted) (left). A stitched overview of the entire nerve section, composed of 10,614 individual images. The boxed region is shown at middle image. The entire area, measuring approximately 1.2 mm^2^, was scanned at 4 nm/pixel in 20 min using a five-point auto-focusing routine (middle). A single hexagonal field of view, composed of 61 images acquired simultaneously (right). A single raw image from the hexagon. Scale bars (left) = 200 μm, (middle) = 30 μm, (right) = 2 μm. SEM, scanning electron microscopy; TEM, transmission electron microscopy.

Electron microscopies are indispensable tools in neuroscience research for observing the ultrastructures of myelin, neurons, and synapses, offering nanoscale resolution beyond the diffraction limit of LMs. The two main EM modalities are scanning EM (SEM) and transmission EM (TEM). SEM generally provides high-resolution 3D surface views by detecting electrons scattered from a sample’s surface. Techniques such as the modified KOH-collagenase (Miller et al., 1982; Ushiki and Ide, 1986, 1987, 1988) and osmium maceration methods (Koga and Ushiki, 2006; Figure 5A) enable unique views of the ultrastructural anatomy of nervous tissue components, including the myelinated and unmyelinated axons, nodes of Ranvier, and surrounding cellular components with their structural associations (Nomura et al., 2013; Bando et al., 2015). TEM is generally employed for 2D imaging by detecting electrons transmitted through ultrathin-sliced (usually 50–80 nm in thickness) samples. The samples are usually embedded within a resin and sectioned using a diamond knife. TEM offers superior resolution and contrast, facilitating detailed visualization of fine intracellular microstructures (Figure 5B). For instance, the periodicity of the multilamellar myelin sheath can be clearly visualized using TEM at magnifications > 50,000x (Figure 5B right). However, TEM imaging is limited to a field of view of a few square millimeters, as the sections are mounted on metal grids with a maximum diameter of 3 mm.

Given the high-resolution capacity of EM with a high degree of precision, appropriate sample preparation, particularly rapid fixation, is crucial to avoid myelin-related artifacts (Wang et al., 2022). In the conventional specimen preparation of nervous tissues, 2.5% glutaraldehyde is typically used for primary fixation, followed by 1% osmium tetroxide for post-fixation. As mentioned earlier, osmium tetroxide effectively fixes the lipid in myelin. Although 0.1 M cacodylate buffer (pH 7.4) is frequently used, phosphate buffer reportedly yields comparable or, sometimes, superior outcomes (Liang et al., 2021). Conventional chemical fixation relies on the diffusion of fixatives, which can induce osmotic changes and shrinkage and thus potentially distort the myelin ultrastructure. High-pressure freezing (HPF) followed by freeze-substitution has emerged as alternative method (Möbius et al., 2016). By physically immobilizing tissue within milliseconds, HPF prevents ice crystal formation, transforming cellular water into an amorphous, glass-like state in a process known as vitrification. This eliminates osmotic artifacts, faithfully retaining axonal circularity and the native compaction of myelin lamellae. A major limitation of this method is that effective vitrification is physically restricted to a depth of approximately 200 μm from the surface. This technique is unsuitable for large tissue volumes because it requires immediate, precise dissection or slicing prior to freezing, which can introduce mechanical damage if not performed with extreme care.

### 4.2 Application of SEM technology for section imaging

Although SEM is traditionally used for surface imaging, it is now frequently applied for the observation of ultrathin sections collected on conductive flat materials such as silicon wafer and glass slide (Figure 5C). Although the resolution of SEM remains lower than that of TEM, this approach offers distinct advantages, including the ability to collect a much larger number of serial section collections, a wider field of view, and greater section durability for repeated observation.

This capacity for large-area imaging is dramatically enhanced by multibeam SEM, which simultaneously utilizes multiple electron beams (61 or 91 beams) to acquire centimeter-scale images at nanometer-scale resolution with unprecedented speed (Eberle et al., 2015). By stitching thousands of high-resolution images, this method provides a seamless view from the tissue level down to the ultrastructural level (Figure 5D). This high-throughput, nanometer-scale imaging capability substantially reduces the time required for large-area acquisition, facilitating brain connectomics research across unprecedented volumes (Shapson-Coe et al., 2024; Wu et al., 2024).

### 4.3 Volume electron microscopy (vEM)

3D-reconstruction volume analysis from serial EM images is a powerful approach for understanding ultrastructural anatomy. In the traditional array-tomography method, manually collected serial ultrathin sections are observed by general TEM or SEM. This method does not require specialized equipment beyond a conventional ultrathin sectioning device and EMs; however, significant technical expertise is required, and the risk of losing sections due to human error remains unavoidable. The automated tape-collecting ultramicrotome (ATUM)-SEM method overcomes this by automatically collecting serial sections onto a conductive tape, greatly simplifying the creation of large section libraries (Schalek et al., 2012; Hayworth et al., 2014). The conductive tape facilitates high-resolution and wide-area imaging by preventing charging. The sections can also be post-stained and re-imaged. This system can reliably collect thousands of serial sections, even at thicknesses of ≤50 nm. This process would be challenging, even for a skilled technician. A disadvantage of this method is that precise computational image alignment of the image series before 3D reconstruction is time-consuming.

Two other techniques, serial block-face (SBF)-SEM and focused ion beam (FIB)-SEM, generate inherently aligned image stacks by iteratively imaging sample block surfaces and subsequently removing a thin layer to expose a new surface. In SBF-SEM, a diamond knife removes layers within the specimen chamber, enabling larger-volume reconstructions exceeding hundreds of micrometers (Denk and Horstmann, 2004). In FIB-SEM, an ion beam mills the surface, offering superior z-resolution in a few nanometers, roughly one-tenth the thickness of a physical ultrathin section, enabling the isotropic analysis (Heymann et al., 2006; Knott et al., 2008; Xu et al., 2017). However, the field of analysis is smaller than that of other methods, practically limited to an area of approximately 100 μm square. A key advantage of SBF- and FIB-SEM is that they can acquire inherently aligned serial z-stack images without loss, greatly simplifying the 3D reconstruction process. However, a common drawback is that tissues are consumed with each imaging cycle. Moreover, preparing samples with optimal conductivity by additional procedures, such as *en bloc* conductive staining, is essential. Recent 3D analysis approaches combining ATUM and FIB efficiently revealed ultrastructures in the targeted area at isotropic resolution (Kislinger et al., 2020). Notably, a region of interest is first identified within the extensive ATUM section library and subsequently targeted for high-resolution, isotropic 3D imaging using FIB-SEM.

Volume EM strategies have been extensively employed in CNS research to resolve complex 3D neural circuits and neural-glial interactions. In the PNS, although the architecture is generally simpler, Schadt et al. (2025) highlighted that 3D volumetric analysis is fundamentally required to resolve the true ultrastructural integrity of the axon-myelin unit, uncovering pathologies such as myelin outfoldings that are ambiguous in 2D cross-sections. In contexts such as nerve regeneration, Leckenby et al. (2019) demonstrated long-distance tracking to capture complex axonal behaviors and trajectories over large spatial ranges. The application of volume EM in the PNS is specifically driven by the need to visualize longitudinal complexities, including pathological ultrastructures and extended regenerative pathways.

### 4.4 Immunoelectron microscopy (iEM)

Immunoelectron microscopy is a powerful technique for elucidating the precise subcellular localization of target molecules within their ultrastructural context, thereby highlighting their specific functions (Nordengen et al., 2015; Weil et al., 2016). The widely applied immuno-gold method visualizes antibody-bound targets as electron-dense nanometer-scale colloidal gold particles, enabling simultaneous observation of the labeled molecule and the surrounding cellular architecture. A major challenge in iEM is the need to preserve both antigenicity and ultrastructures, leading to the development of several protocols. In the pre-embedding protocol, target antigenicity is well-preserved because the immunoreaction is processed before the typical EM protocol, including osmium fixation, dehydration, and embedding that may denature antigens (Polishchuk and Polishchuk, 2019; Tao-Cheng et al., 2021). Ultrastructures are fairly well-preserved if permeabilization is adequately mild, while excessive treatment easily causes the artificial collapse of many structures, such as plasma membranes comprising myelin lamellae. The post-embedding protocol is excellent for preserving ultrastructure, as samples undergo full EM preparation for hydrophilic resin embedding before immunolabeling. Loss or masking of antigens during the harsh embedding process often greatly reduces labeling efficiency. Samples for these two methods are typically prepared using standard EM protocols with modifications for IHC. Conversely, the cryosection iEM protocol such as the Tokuyasu method avoids resin embedding, effectively resolving the trade-off between ultrastructural preservation and antigenicity (Tokuyasu, 1973; Möbius and Posthuma, 2019). Additionally, by using high-pressure freezing to vitrify samples, this approach minimizes chemical cross-linking and dehydration and thereby preserves antigenicity (van Donselaar et al., 2007). Furthermore, this approach allows for efficient labeling within a native-like myelin architecture free from shrinkage artifacts. However, this technique requires specialized equipment for sample preparation, including a high-pressure freezer and cryo-ultramicrotome. Furthermore, different from pre- and post-embedding protocols, the cryo-approach necessitates a dedicated workflow starting from fresh tissue; thus, it cannot be applied retrospectively to conventionally fixed samples.

### 4.5 Correlative light and electron microscopy (CLEM)

Correlative light and electron microscopy combines the advantages of LMs and EMs, allowing the mapping of molecular or functional information with ultrastructural context. While iEM visualizes target molecules directly with nanometer precision, it is essentially EM restricted to small fields of view. The workflow leverages the primary strength of LM, which is the ability to identify specific, fluorescently-labeled structures across large fields of view even under physiological conditions, to guide subsequent EM imaging of the same region of interest. Although iEM and CLEM are distinct approaches, the use of fluorescence-colloidal gold dual-conjugated antibodies can effectively bridge these approaches. This specific method allows researchers to identify regions of interest via fluorescence in the microscale field of LM and to subsequently visualize the direct localization of target molecules via gold particles in EM, thereby utilizing iEM and CLEM in a complementary manner.

A distinct advantage of CLEM, particularly when using genetically encoded fluorescent markers to label specific cells or organelles, is the superior preservation of ultrastructure. Unlike pre-embedding immunolabeling, this approach eliminates the need for antibody reactions, rendering harsh treatments such as antigen retrieval or permeabilization unnecessary.

During CLEM process, the fluorescence quenching that occurs during the preparation of the electron microscopy (EM) sample, including osmium fixation and resin embedding, poses a persistent challenge, particularly for conventional antibody-based staining. In order to address this challenge, the utilization of osmium-resistant fluorescent proteins (Tanida et al., 2020) and proximity labeling with biotin ligases (Sanada et al., 2022) enables “in-resin CLEM,” a technique designed to retain fluorescence signals even after full EM processing. These advancements also suggest a potential solution for a technical challenge in conventional CLEM: the accurate correlation of images due to the discrepancy in z-axial resolution between LM and EM. While simple 2D overlays are often insufficient for identifying small, sub-micron structures, and precise correlation typically requires complex 3D correlation strategies, in-resin CLEM offers a compelling alternative strategy. By detecting fluorescence directly from the ultrathin sections for EM, this method theoretically eliminates the z-axis mismatch, facilitating precise superposition.

The EM component of a CLEM experiment can employ several optional methods depending on the aims of the investigation. The multibeam SEM is a method that facilitates CLEM workflows across areas spanning several square centimeters (Shibata et al., 2019). 3D-CLEM extends this approach to volume imaging, enabling the analysis of complex, fluorescently-labeled neurons and axons (Luckner et al., 2018; Booth et al., 2019). This approach enables powerful quantitative analyses by correlating functional or molecular signals from LM with high-resolution 3D morphological data from EM.

## 5 Quantitative analysis for myelin

Quantitative analysis of axon imaging data can encompass molecular profiling via techniques such as IHC and structural morphometry. This section focuses on the latter, the quantitative measurement of physical features. For example, the number and density of myelinated axons can be assessed using bright-field LMs, whereas dark-field LMs offer the necessary contrast to readily quantify smaller-caliber and non-myelinated axons. For the myelin morphometry, the g-ratio, the ratio of the axon diameter to the total outer diameter of the myelin, originated from Schmitt and Bear (1937), is frequently analyzed (Boullerne, 2016). This morphometrical parameter is functionally relevant because myelin thickness strongly influences axonal conduction velocity. In the 2D analysis, as axons appear not always circular but instead as ovals, shape adjustment steps such as approximation are often applied (Moiseev et al., 2019; Bartmeyer et al., 2021).

The g-ratio has traditionally been calculated based on EM data, while recent MRI-based approaches have extended such measurements to considerably larger areas (Campbell et al., 2018; Mohammadi and Callaghan, 2021; Grouza et al., 2024). However, the accuracy and comparability of MRI-based g-ratio assessment remain subjects of critical debate. Unlike conventional 2D EM, measuring individual axons at the micrometer scale, MRI-derived g-ratios provide an aggregate measure within a voxel. These values are highly dependent on the specific myelin-sensitive measures and employed biophysical models (Berg et al., 2022; Mohammadi and Callaghan, 2021). For instance, Berg et al. (2022) reported that g-ratio values varied significantly across different MRI techniques, particularly in pathological conditions such as multiple sclerosis lesions where tissue complexity is high. Furthermore, histological validation studies have highlighted inherent biases. West et al. (2018) demonstrated that while the MRI g-ratio correlates with histological measures, it often exhibits bias owing to the presence of non-myelinated axons. These axons are considered in diffusion-based axonal volume fractions (AVF) but do not contribute to myelin-sensitive MRI metrics, leading to an overestimation of the aggregate g-ratio because non-myelinated axons effectively act as components with a g-ratio of 1. Other tissue features, such as fiber density and crossing fiber orientations, also impact the estimation of volume fractions, potentially leading to over-estimations or under-estimations of the aggregate g-ratio (Stikov et al., 2015; Berg et al., 2022). Therefore, while MRI-based approaches offer non-invasive longitudinal insights, standardized protocols and cautious interpretation are essential when evaluating complex tissue environments. For instance, parameters derived from q-space diffusion MRI have demonstrated strong correlations with myelin content in the brain and spinal cord, supporting their utility in assessing white matter pathology (Fujiyoshi et al., 2016). The validation of MRI-based metrics against gold-standard histology is critical for accurate interpretation.

Recent progress in computational image analysis has revolutionized myelin morphometry, offering solutions tailored to specific imaging modalities and research needs. In LMs, which remain the standard for high-throughput diagnostic screening, automated pipelines have been developed to facilitate rapid fiber quantification and g-ratio analysis (Thomson et al., 2023; Lloyd et al., 2024). Recent iterations emphasize user-friendly interfaces to broaden accessibility (Suchyta et al., 2025) and employ generative models to enable stain-free histomorphometry (Coto Hernández et al., 2022a). For 2D electron microscopy, deep learning-based tools have standardized ultrastructural segmentation (Zaimi et al., 2018), while specialized machine-learning workflows allow for the intricate analysis of sub-compartments such as the inner tongue (Carrillo-Barberà et al., 2023). Furthermore, addressing the complexity of vEM, advanced pipelines (Abdollahzadeh et al., 2019, 2021) have been developed to tackle the dense instance segmentation required for large-scale volumetric datasets (Schadt et al., 2025). Regional segmentation has long been a major bottleneck in analysis, particularly for the massive datasets acquired for vEM. The widespread adoption of these rapidly developing methods has the potential to resolve this longstanding challenge.

Currently, the field is advancing toward AI-driven multimodal data fusion. Building on foundational strategies for integrating diverse neuroimaging datasets (Zhang et al., 2020), emerging computational frameworks now bridge the gap between molecular omics and multiscale imaging. For instance, graph representation learning allows for the joint embedding of spatial transcriptomics and epigenomics to infer cross-modality regulatory relationships within the tissue microenvironment (Miao et al., 2025). Furthermore, deep-learning approaches are increasingly utilized to cross-validate non-invasive imaging biomarkers with underlying molecular signatures, providing a more holistic understanding of myelin biology. These integrative approaches have the potential to reveal complex structure-function relationships that remain inaccessible through single-modality analysis, thereby supporting precision medicine in the future (Huang and Shu, 2025).

## 6 Discussion

The various approaches for biological sample imaging have advanced with remarkable innovations and improvements in imaging modalities, specimen preparation techniques, and data calculation technology. Moreover, advancements in computational technology have contributed to the analysis of large data sets, including 3D reconstructed structures. Each imaging approach has its own characteristics—including applicability, spatial resolution, and sample size—determining its unique strengths and limitations. Recent macro- to meso-scale imaging modalities such as MRI, PET, and X-ray CT provide *in vivo* or non-destructive imaging at extraordinarily large scales, including whole human organs with higher sensitivity, allowing even calculation of myelin thickness. These techniques are limited in their ability to visualize microarchitectural details. Microscale imaging techniques, including traditional histology with widefield microscopy, offer a variety of information ranging from whole rodents’ organs down to the subcellular level, based on a solid historical foundation. Various advanced LMs have enhanced our understanding of the relationships between cellular structures and their molecular changes, owing to their broader applicability and capabilities for imaging large areas, *in vivo* dynamics, or achieving super-resolution. However, achieving the highest resolution with many advanced LMs often requires focusing on a target structure. Nanoscale imaging devices, EMs, provide valuable morphological information at an ultrastructural level, which is unattainable by other modalities. Their limitations include a limited sample size, typically only a few millimeters in maximum, and the need for strong fixation during sample preparation. Methods including iEM and CLEM strategies are particularly powerful to directly link molecular profiles to ultrastructural morphology. Ultimately, the future of myelin research lies in integrating imaging toolkits to achieve a more holistic understanding of myelin profiles in health and disease. Through the lens of this integrated imaging, the full spectrum of glial biology can be revealed—from the visualization of developmental processes and functional dynamics to the detailed characterization of pathophysiological changes—thereby uncovering novel targets for therapeutic intervention.
